# Ionic mitigation of CD4^+^ T cell metabolic fitness, Th1 central nervous system autoimmunity and Th2 asthmatic airway inflammation by therapeutic zinc

**DOI:** 10.1038/s41598-022-04827-6

**Published:** 2022-02-04

**Authors:** Anna Krone, Yan Fu, Simon Schreiber, Johanna Kotrba, Loisa Borde, Aileen Nötzold, Christoph Thurm, Jonas Negele, Tobias Franz, Sabine Stegemann-Koniszewski, Jens Schreiber, Christoph Garbers, Aniruddh Shukla, Robert Geffers, Burkhart Schraven, Dirk Reinhold, Anne Dudeck, Annegret Reinhold, Andreas J. Müller, Sascha Kahlfuss

**Affiliations:** 1 Institute of Molecular and Clinical Immunology, Medical Faculty, Otto-von-Guericke University Magdeburg, Magdeburg, Germany; 2 Experimental Pneumology, Department of Pneumology, University Hospital Magdeburg/Medical Faculty, Otto-von-Guericke-University, Magdeburg, Germany; 3 Institute of Pathology, Medical Faculty, Otto-von-Guericke University Magdeburg, Magdeburg, Germany; 4 Institute of Medical Microbiology and Hospital Hygiene, Medical Faculty, Otto-von-Guericke University Magdeburg, Magdeburg, Germany; 5 Genome Analytics, Helmholtz-Center for Infection Research (HZI), Braunschweig, Germany; 6 Intravital Microscopy of Infection and Immunity, Helmholtz-Center for Infection Research (HZI), Braunschweig, Germany; 7 Health Campus Immunology, Infectiology and Inflammation (GCI3), Medical Faculty, Otto-Von-Guericke University Magdeburg, Magdeburg, Germany; 8 Center for Health and Medical Prevention (CHaMP), Otto-von-Guericke-University, Magdeburg, Germany

**Keywords:** Biomarkers, Adaptive immunity, Applied immunology, Autoimmunity, Cell death and immune response, Immune cell death, Lymphocytes, Signal transduction

## Abstract

T helper (Th) cells provide immunity to pathogens but also contribute to detrimental immune responses during allergy and autoimmunity. Th2 cells mediate asthmatic airway inflammation and Th1 cells are involved in the pathogenesis of multiple sclerosis. T cell activation involves complex transcriptional networks and metabolic reprogramming, which enable proliferation and differentiation into Th1 and Th2 cells. The essential trace element zinc has reported immunomodulatory capacity and high zinc concentrations interfere with T cell function. However, how high doses of zinc affect T cell gene networks and metabolism remained so far elusive. Herein, we demonstrate by means of transcriptomic analysis that zinc aspartate (UNIZINK), a registered pharmaceutical infusion solution with high bioavailability, negatively regulates gene networks controlling DNA replication and the energy metabolism of murine CD3/CD28-activated CD4^+^ T cells. Specifically, in the presence of zinc, CD4^+^ T cells show impaired expression of cell cycle, glycolytic and tricarboxylic acid cycle genes, which functionally cumulates in reduced glycolysis, oxidative phosphorylation, metabolic fitness and viability. Moreover, high zinc concentrations impaired nuclear expression of the metabolic transcription factor MYC, prevented Th1 and Th2 differentiation in vitro and reduced Th1 autoimmune central nervous system (CNS) inflammation and Th2 asthmatic airway inflammation induced by house dust mites in vivo. Together, we find that higher zinc doses impair the metabolic fitness of CD4^+^ T cells and prevent Th1 CNS autoimmunity and Th2 allergy.

## Introduction

T cell proliferation and differentiation into T helper (Th) cells is crucial for immunity against pathogens. While Th1 cells mediate immunity to viruses and intracellular bacteria, Th2 cells provide immunity against parasites such as helminths. However, Th1 and Th2 cells are also involved in autoimmune diseases and in the development of allergies, respectively. Here, Th1 cells (besides Th17 cells) contribute to autoimmune diseases, like e.g. multiple sclerosis (MS), and Th2 cells mediate allergic asthma^1^, which is frequently evoked by house dust mites^2^. It is of note that allergic asthma and autoimmune diseases show an increasing incidence especially in western civilizations^2–4^. This necessitates the development of novel and more efficient therapeutics with no or only little side effects besides currently existing treatment regimens for these diseases.

The bivalent cation zinc is an essential trace element and was shown to affect innate and adaptive immune responses^5–9^. Zinc administration to humans was reported to be safe with no significant side effects observed so far^10–14^. High dose zinc supplementation impairs CD4^+^ T cell activation, proliferation and viability, and zinc is discussed as treatment option for autoimmune diseases and allergy^15–20^. However, it is yet unknown in which way high zinc concentrations modulate transcriptional gene networks in CD4^+^ T cells and how this is related to its immunosuppressive effect on these cells. In addition, it was not yet investigated whether and how higher zinc concentrations interfere with the metabolism of CD4^+^ T cells, although metabolic reprogramming is an important prerequisite for T cell activation, proliferation, and their differentiation into distinct Th populations such as Th1 and Th2 cells^21–24^.

## Results

### High dose zinc negatively regulates cell cycle genes in CD4^+^ T cells

Zinc has immunomodulatory capacity through its action on innate and adaptive immune cells including T cells. Especially high doses of zinc impair CD4^+^ T cell activation and proliferation^9,15,16,20,25^. In this context, zinc is discussed as additional therapy regimen for autoimmune diseases and allergies including asthma^17–19^. It is however unclear how higher zinc concentrations change transcriptional networks and the metabolic state of T cells after activation.

**Figure 1:**
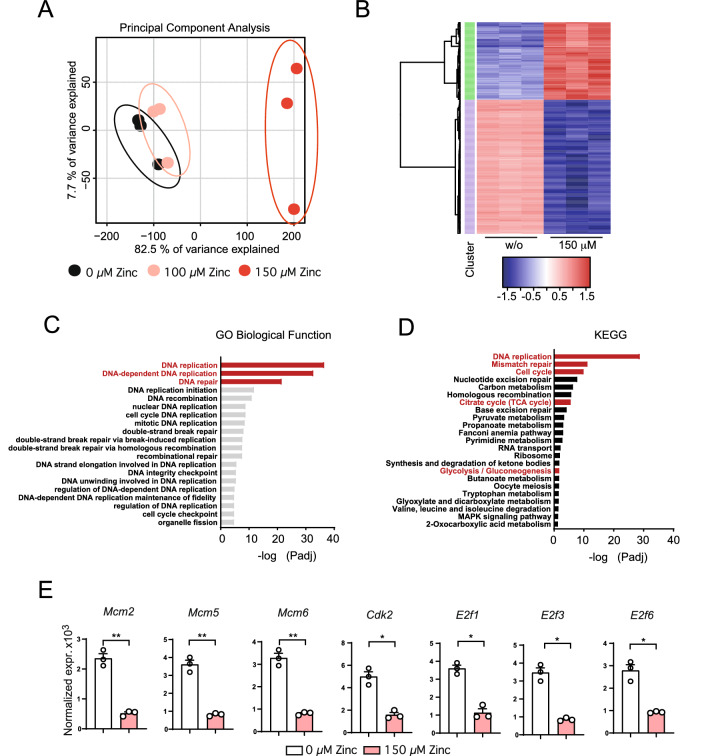
High dose zinc aspartate negatively regulates genes controlling cell cycle entry and progression in CD4^+^ T cells. (**A**) Principal component analysis (PCA) of the individual biological samples used for bulk RNA sequencing. (**B**) Unsupervised clustering heatmap showing differentially expressed genes (DEG) between individual biological samples. (**C**) TOP Gene ontology (GO) Biology Function pathways based on p-value. Shown is the –log_10_ of the p-values of the individual pathways. (**D**) TOP KEGG pathways based on p-value. Shown is the –log_10_ of the p-values of the individual pathways. (**E**) Normalized gene expression of *Mcm2*, *Mcm5*, *Mcm6*, *Cdk2*, *E2f1*, *E2f3*, and *E2f6* determined by RNA sequencing. Statistical analysis in (**E**) by unpaired student’s t test. Dots represent individual mice used to isolate OT-II TCR tg CD4^+^ T cells. RNA sequencing data was generated in biological triplicates from 3 mice. KEGG was used for pathway analysis^26^. p* < 0.05, **p < 0.01, ***p < 0.001.

In order to investigate this question, we set out to determine the effect of high zinc concentrations on the transcriptome of activated T cells in vitro. To this end, we stimulated CD4^+^ OT-II T cell receptor (TCR) transgenic (tg) T cells with CD3/CD28 in the absence or presence of 100 μM or 150 μM zinc aspartate (UNIZINK) for 24 h followed by bulk RNA sequencing. We decided to use 100 μM and 150 μM zinc aspartate, because at these concentrations T cells start to be functionally compromised as indicated by reduced activation and impaired proliferation in own pilot experiments and based on published studies^25^. Principal component analysis (PCA) revealed that activated CD4+ T cells in the presence of either 100 μM or 150 μM zinc aspartate separated from activated T cells that have not been treated (Fig. 1A, Suppl. Fig. S1A). CD4^+^ T cells incubated with 150 μM zinc aspartate discriminated the most from the other two conditions in terms of their transcriptome and differentially expressed genes (DEGs). T cells treated with 150 μM zinc aspartate showed both significantly up- and downregulated genes (Fig. 1B). To analyze which gene networks are mostly affected by the addition of exogenous high zinc doses we performed an unbiased pathway analysis of CD4^+^ T cells in the presence of 150 μM zinc aspartate using Gene Ontology (GO) and KEGG. Our analysis revealed especially pathways related to cell cycle as well as DNA replication in CD4^+^ T cells to be most significantly regulated by 150 μM zinc aspartate (Fig. 1C,D). Pathways related to an acute heavy metal stress responses were not detected. Within the top three KEGG pathways ‘DNA replication’, ‘Mismatch repair’ and ‘Cell cycle’ we, however, detected several genes encoding for cell cycle regulators to be significantly downregulated in activated CD4^+^ T cells upon 150 μM zinc including *Mcm2*, *Mcm5*, *Mcm6*, *Cdk2*, *E2f1*, *E2f3*, and *E2f6* (Fig. 1E). Of note, all of these genes have been directly linked to cell cycle entry, progression or regulation (Suppl. Fig. S1B)^27–29^.

### High concentrations of zinc inhibit blast formation, activation, proliferation and the metabolic fitness of CD4^+^ T cells

**Figure 2:**
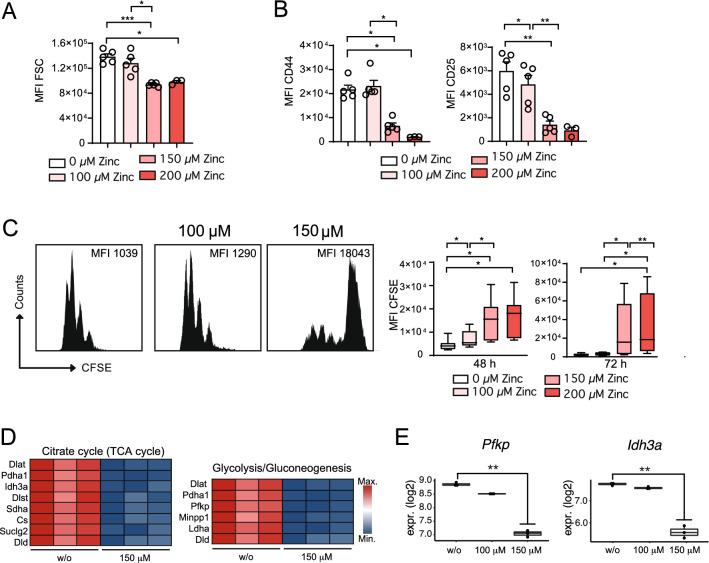
High concentrations of zinc inhibit blast formation, activation, proliferation and the metabolic fitness of CD4^+^ T cells. (**A**) Blast formation of OT-II TCR tg CD4^+^ T cells in the absence and presence of 100 μM, 150 μM, and 200 μM zinc aspartate analyzed by determining the FSC using FACS after 24 h. (**B**) MFI of CD44 and CD25 expression of OT-II TCR tg CD4^+^ T cell activation after 48 h. (**C**) CFSE dilution of OT-II TCR tg CD4^+^ T cells in the absence and presence of 100 μM, 150 μM, and 200 μM zinc aspartate after 72 h of stimulation. (**D**) Heatmap showing significant genes out of the KEGG pathways ‘Glycolysis/Gluconeogenesis’ and ‘Citrate cycle (TCA cycle)’ identified in Fig. 1D. (**E**) Normalized gene expression of *Pfkp* and *Idh3a* determined by RNA sequencing. Statistical analysis in (**A**–**C**) by one-way ANOVA followed by Tukey’s honestly significant difference (HSD) post hoc test for multiple comparisons. Data in (**A**–**C**) are from 3 experiments and dots represent individual mice used to isolate OT-II TCR tg CD4^+^ T cells. Data in (**C**) are from 8 mice per group. RNA sequencing data in (**D**,**E**) was generated in biological triplicates from 3 mice. KEGG was used for pathway analysis^26^. *p < 0.05, **p < 0.01, ***p < 0.001.

In line with our transcriptome analysis, CD4^+^ OT-II TCR tg T cells stimulated with CD3/CD28 were inhibited in blast formation (Fig. 2A), activation as reflected by reduced CD44 and CD25 expression (Fig. 2B) and proliferation (Fig. 2C) in the presence of 100 μM and 150 μM (as well as 200 μM) zinc aspartate. Pathway analysis had demonstrated that zinc negatively regulates several genes related to cell cycle control and DNA replication in CD4^+^ T cells. Cell cycle entry depends on the generation of sufficient building blocks for consecutive proliferation, which is guaranteed through metabolic reprogramming in T cells^30^. Metabolic reprogramming is characterized through an upregulation of genes encoding for enzymes of glycolysis to feed various metabolic pathways such as the tricarboxylic acid (TCA) cycle with metabolic intermediates^23^. In this context, KEGG pathway analysis also identified the pathways ‘Glycolysis/Gluconeogenesis’ and ‘Citrate cycle (TCA cycle)’ to be among the most significantly affected pathways following activation in the presence of zinc (pl. see Fig. 1). Of note, within these pathways we detected several glycolysis and TCA cycle genes (Fig. 2D) to be significantly downregulated in CD4^+^ T cells in the presence of 150 μM zinc aspartate, including *Pfkp* and *Idh3a* (Fig. 2E), which encode for key and rate-limiting enzymes of these metabolic pathways, respectively. Of note, in the presence of high zinc aspartate concentrations gene expression of metabolic genes and regulators including *Myc* were already impaired 6 h after stimulation (Suppl. Fig. S1C) at time points, at which cells had not undergone early apoptosis as determined by AnnexinV staining (Suppl. Fig. S1D) and as reported earlier^25^. In line with the latter, 100 μM zinc had reduced the absolute numbers of viable cells at 24 h to only 94 ± 12% and 150 μM zinc to only 93 ± 28% compared to stimulated but untreated cells (0 μM zinc). Following 48 h of stimulation, 100 μM zinc had reduced the absolute numbers of viable cells to 91 ± 17% and 150 μM zinc to 77 ± 38%. At 48 h, CD4^+^ T cells showed reduced viability at higher zinc concentrations (Suppl. Fig. S1E) confirming previous findings^25^.

**Figure 3:**
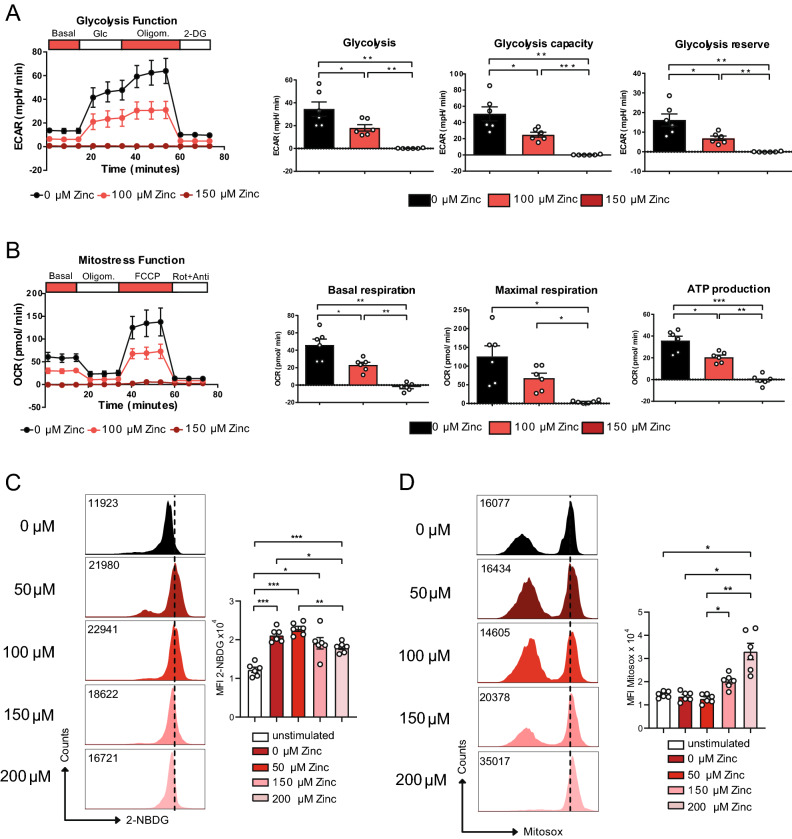
Zinc aspartate reduces glycolysis and OXPHOS in CD4^+^ T cells. (**A**) Extracellular acidification rate (ECAR) of OT-II TCR tg CD4^+^ T cells in the absence and presence of 100 and 150 μM zinc aspartate after 24 h of stimulation and adding glucose, oligomycin, and 2-DG. Bar graphs show glycolysis, glycolysis capacity, and glycolysis reserve. (**B**) Oxygen consumption rate (OCR) of OT-II TCR tg CD4^+^ T cells in the absence and presence of 100 and 150 μM zinc aspartate after 24 h of stimulation and adding oligomycin, FCCP, and rotenone/antimycin. Bar graphs show basal respiration, maximal respiration and ATP production. (**C**) Glucose uptake determined using 2-NBDG and measured by FACS for the indicated groups. (**D**) Mitochondrial ROS production determined using Mitosox and measured by FACS for the indicated groups. Statistical analysis in (**A**–**D**) by one-way ANOVA followed by Tukey’s honestly significant difference (HSD) post hoc test for multiple comparisons. Data in (**A**–**D**) are from 3 experiments. Dots represent individual mice used to isolate OT-II TCR tg CD4^+^ T cells. *p < 0.05, **p < 0.01, ***p < 0.001.

To test whether the decrease in metabolic gene expression of glycolysis and TCA cycle genes upon zinc would also have functional consequences, we measured extracellular acidification rate (ECAR) and oxygen consumption rate (OCR) to determine glycolysis and oxidative phosphorylation (OXPHOS) of CD4^+^ OT-II TCR tg T cells after CD3/CD28 stimulation in the absence and presence of zinc. Seahorse analysis showed that CD4^+^ T cells in the presence of 100 μM and 150 μM zinc aspartate were significantly inhibited in conducting glycolysis (Fig. 3A) and OXPHOS (Fig. 3B). In line with reduced glycolysis also glucose uptake of CD4^+^ OT-II TCR tg T cells stimulated by CD3/CD28 was inhibited by high zinc concentrations (Fig. 3C). In contrast, mitochondrial reactive oxygen species (ROS) production appeared significantly increased at these concentrations (Fig. 3D).

### Zinc inhibits MYC as a transcriptional regulator of metabolic gene expression in CD4^+^ T cells

**Figure 4:**
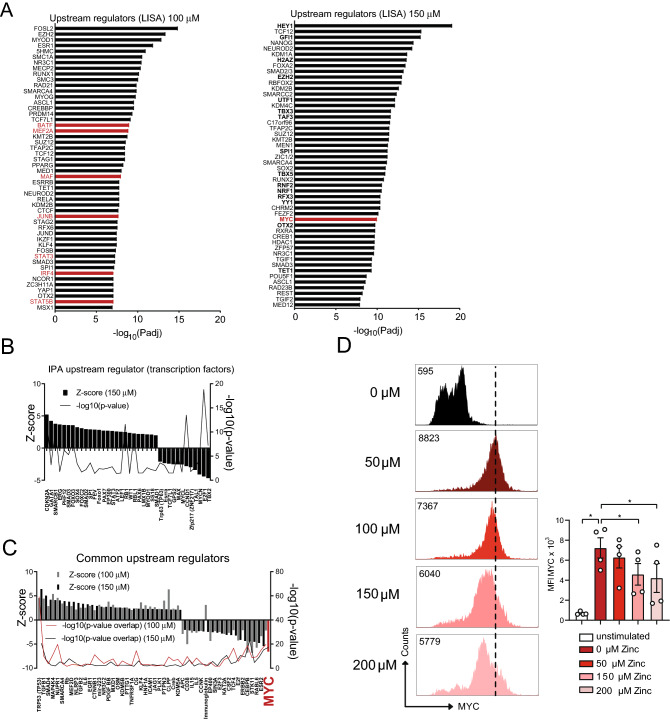
High zinc concentrations inhibit MYC as a transcriptional regulator of metabolic gene expression in CD4^+^ T cells. (**A**) Upstream regulator anaylsis using Landscape in silico analysis (Lisa; http://lisa.cistrome.org/)^31^ of the RNA sequencing data from OT-II TCR tg CD4^+^ T cells in the presence of 100 μM or 150 μM zinc aspartate 24 h after activation. (**B**) Upstream regulators specific to OT-II TCR tg CD4^+^ T cells in the presence of 150 μM zinc aspartate identified by IPA. (**C**) Shared upstream regulators between OT-II TCR tg CD4^+^ T cells in the presence of 100 μM and 150 μM zinc aspartate identified by Ingenuity Pathway Analysis (IPA). (**D**) Histograms show nuclear MYC expression in OT-II CD4^+^ T cells activated for 24 h in the absence or presence of the indicated zinc concentrations. Graphs show cumulated data from 4 mice. RNA sequencing data (**A**–**C**) was generated in biological triplicates from 3 mice. Statistical analysis in (**D**) by one-way ANOVA followed by Tukey’s honestly significant difference (HSD) post hoc test for multiple comparisons. Data in (**D**) are from 4 mice. *p < 0.05, **p < 0.01, ***p < 0.001.

In order to identify the key upstream transcription factors of the metabolic and cell cycle genes regulated by zinc we performed epigenetic Landscape In Silico deletion Analysis (Lisa)^31^ and used Ingenuity Pathway Analysis (IPA). Using Lisa, we detected several transcription factors important for T cell function in general such as BATF, MEF2, MAF, JUNB, IRF4, STAT3 and STAT5 to be regulated by high zinc concentrations (Fig. 4A), which was in line with reduced activation of CD4^+^ T cells in the presence of 100 μM and 150 μM zinc aspartate. Interestingly, we also identified the transcription factor MYC, a key regulator of metabolic and cell cycle gene transcription, to be among the top upstream regulators of the genes regulated by high doses zinc aspartate (Fig. 4A–C). Of note, the transcription factor FOXO1 was recently shown to be a negative regulator of glycolysis and T cell fitness^32,33^ and appeared upregulated upon zinc (Fig. 4B). MYC, in contrast, is a critical transcription factor and positive regulator involved in the upregulation of cell cycle and glycolysis genes during metabolic reprogramming following initial T cell activation^34,35^ and appeared to be the top upstream regulator significantly downregulated in the presence of both 100 μM and 150 μM zinc (Fig. 4C). To validate these findings on the protein level we measured nuclear MYC protein expression in CD4^+^ OT-II TCR tg T cells stimulated with CD3/CD28 in the absence or presence of increasing concentrations of zinc aspartate (UNIZINK) after 12 and 24 h. MYC protein expression appeared significantly inhibited by increasing zinc aspartate concentrations (Fig. 4D). Taken together our results indicated that high dose zinc negatively regulates metabolic reprogramming, glycolysis, cell cycle entry and metabolic fitness through controlling gene networks including metabolic genes regulated by MYC in CD4^+^ T cells.

### Zinc aspartate prevents Th1 CNS autoimmunity and Th2 asthmatic airway inflammation induced by house dust mites

**Figure 5:**
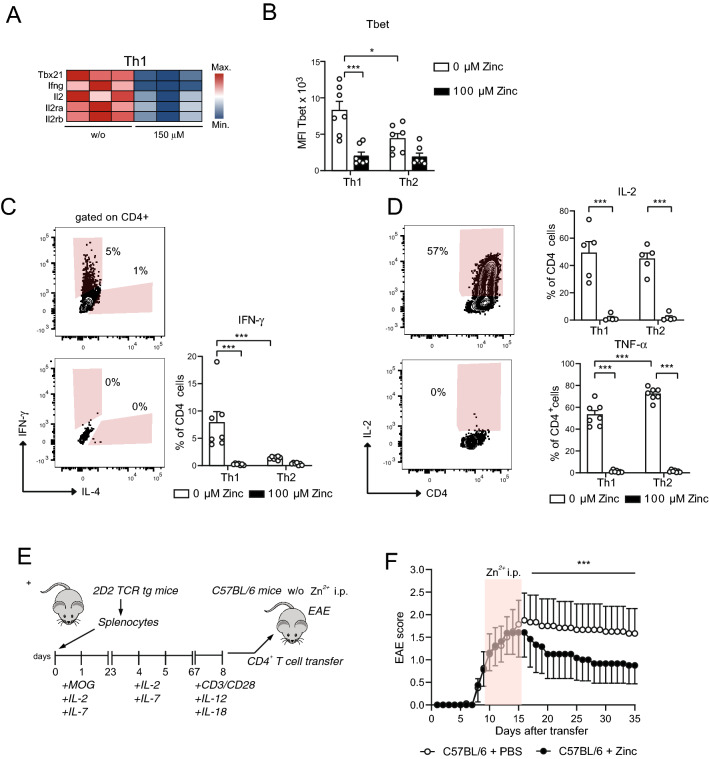
Zinc aspartate prevents Th1 CNS autoimmunity. (**A**) Heatmap showing Th1 genes of OT-II TCR tg CD4^+^ T cells in the absence and presence of 150 μM zinc aspartate. (**B**) MFI of Tbet measured by FACS of OT-II TCR tg CD4^+^ T cells differentiated into Th1 and Th2 cells in the absence and presence of 100 μM zinc aspartate. (**C**) Frequencies of CD4^+^IFN-γ^+^ cells measured by FACS after Th1 and Th2 differentiation in the absence and presence of 100 μM zinc aspartate. (**D**) Frequencies of CD4^+^IL-2^+^ and CD4^+^TNF-α^+^ cells measured by FACS after Th1 and Th2 differentiation in the absence and presence of 100 μM zinc aspartate. (**E**) Splenic T cells of 2D2 mice were stimulated in vitro with MOG_35-55_ in the presence of IL-2 and IL-7 and were reactivated with plate bound anti-CD3 and anti-CD28 in the presence of IL-12 and IL-18. Fully activated transgenic 2D2 T cells were adoptively transferred into C57BL/6 recipients. Zinc aspartate was administered daily i.p. (15 µg/animal) starting at day 8 after the appearance of first clinical signs. Zinc treatment was continued for 7 days. The control group received PBS. (**F**) The clinical score of passive EAE was assessed for 35 days after transfer. Data are shown as mean ± SEM. RNA sequencing data in (**A**) was generated in biological triplicates from 3 mice. Statistical analysis in (**B**–**D**) by unpaired student’s t test and in (**F**) by non-parametric Wilcoxon matched pairs test. Dots represent individual mice used to isolate OT-II TCR tg CD4^+^ T cells for Th differentiation. Data in (**B**–**D**) are from 3 experiments. Data in (**F**) are from 8 mice per group. *p < 0.05, **p < 0.01, ***p < 0.001.

Transcriptomics of in vitro CD3/CD28 activated CD4^+^ OT-II TCR tg T cells had shown that zinc regulates several genes related to cell cycle regulation and energy metabolism. These genes were part of several KEGG pathways including ‘DNA replication’, ‘Oxidative phosphorylation’, ‘Fatty acid elongation’, ‘mTOR signaling pathway’, ‘AMPK signaling pathway’, or ‘PPAR signaling pathway’ (Suppl. Fig. S1F). Many of these pathways appeared interconnected in the KEGG network indicating that they indeed share to a significant degree the same genes regulated by zinc. These results were furthermore in line with impaired glycolysis and OXPHOS cumulating in reduced activation and proliferation upon zinc. In KEGG network analysis we also detected the pathways ‘Th1 and Th2 cell differentiation’ and ‘Cytokine-cytokine receptor interaction’. Among the most downregulated genes in the ‘Th1 and Th2 cell differentiation’ KEGG pathway were several genes important for Th1 differentiation and function such as *Tbx21*, *Ifng*, *Il2*, *Il2ra*, and *Il2rb* (Fig. 5A). In line with this, in vitro differentiation of CD4^+^ OT-II TCR tg T cells into Th1 cells in the presence of high zinc aspartate concentrations was significantly impaired indicated by reduced expression of the Th1 transcription factor Tbet (Fig. 5B) and the Th1 signature cytokine IFN-γ (Fig. 5C) as well as of the cytokines IL-2 and TNF-α (Fig. 5D). Th1 cells are main effector cells involved in the pathogenesis of MS. To test whether zinc aspartate would also affect Th1 autoimmunity in vivo, we induced experimental autoimmune encephalitis (EAE), a mouse model for MS, by stimulating 2D2 TCR tg CD4^+^ T cells with MOG_35-55_ peptide in vitro, differentiating them into Th1 cells and transferring them into C57BL/6 recipient mice (Fig. 5E). In vitro-activated MOG-specific CD4^+^ T cells showed a high expression of CD69 and low expression of CD62L indicative of effective activation before T cell transfer (Suppl. Fig. S1G). After transfer of the 2D2 TCR tg donor CD4^+^ T cells into C57BL/6 mice part of the recipient mice were i.p. treated with zinc aspartate from day 8 after transfer with appearance of clinical EAE signs until day 15. Importantly, mice that received zinc aspartate showed a significantly impaired disease score compared to recipient mice, which only received PBS as control (Fig. 5F).

**Figure 6:**
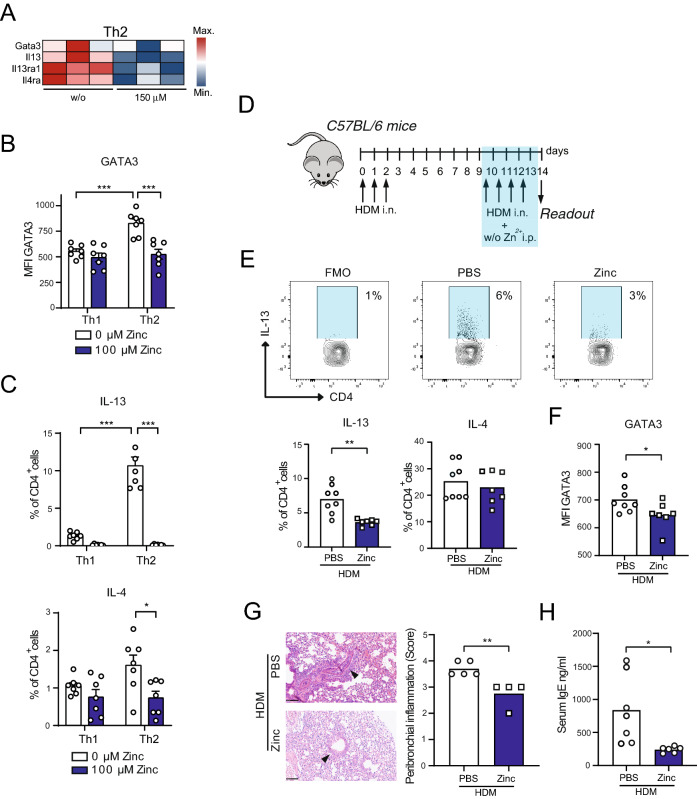
Zinc impairs Th2 house dust mite-induced asthmatic airway inflammation. (**A**) Heatmap showing Th2 genes of OT-II TCR tg CD4^+^ T cells in the absence and presence of 150 μM zinc. (**B**) MFI of GATA3 measured by FACS of OT-II TCR tg CD4^+^ T cells differentiated into Th1 and Th2 cells in the absence and presence of 100 μM zinc aspartate. (**C**) Frequencies of CD4^+^IL-13^+^ and CD4^+^IL-4^+^cells measured by FACS after Th1 and Th2 differentiation in the absence and presence of 100 μM zinc aspartate. (**D**) Mouse model of Th2 asthmatic airway inflammation induced by house dust mite (HDM) extract. (**E**) Frequencies of lung CD4^+^IL-13^+^ and CD4^+^IL-4^+^cells measured by FACS in the mouse model shown in Fig. 3D at day 14. (**F**) MFI of GATA3 in lung CD4^+^ T cells measured by FACS in the mouse model shown in Fig. 3D at day 14. (**G**) Peribronchial inflammation in the lungs from mice of the HDM asthma model shown in Fig. 3D ad day 14. (**H**) Total IgE serum levels of mice from the HDM asthma model shown in Fig. 3D ad day 14. RNA sequencing data in (**A**) was generated in biological triplicates from 3 mice. Statistical analysis in in (**B**) and (**C**) and (**E**–**H**) by unpaired student’s t test. Dots represent individual mice. Data are from 3 (**B**,**C**) and 2 (**E**–**H**) experiments. *p < 0.05, **p < 0.01, ***p < 0.001.

Among the most significantly downregulated genes in the ‘Th1 and Th2 cell differentiation’ KEGG pathway we also detected transcription factors and signature cytokines of Th2 differentiation and function including *Gata3*, *Il13*, *Il13ra1* and *Il4ra* (Fig. 6A). In line with this, in vitro differentiation of CD4^+^ OT-II TCR tg T cells into Th2 cells in the presence of zinc aspartate was inhibited as reflected by significantly reduced expression of the master Th2 transcription factor GATA3 (Fig. 6B) and the Th2 cytokines IL-4 and IL-13 (Fig. 6C) in the presence of high zinc concentrations. To test whether zinc affects Th2 allergy in vivo, we applied a mouse model of allergic asthma, in which C57BL/6 mice underwent intranasal (i.n.) sensitizations and rechallenges with house dust mite (HDM) extract that induces strong and robust Th2 asthmatic airway inflammation (Fig. 6D). Part of the C57BL/6 mice received i.p. injections with zinc aspartate. FACS analysis at day 14 of the model revealed that mice receiving i.p. zinc injections showed significantly lower frequencies of IL-13 producing CD4^+^ T cells in their lungs (Fig. 6E). Furthermore, GATA3 expression was significantly impaired in lung CD4^+^ T cells isolated from mice that were injected with zinc (Fig. 6F). Zinc administration to C57BL/6 mice also prevented from peribronchial asthmatic airway inflammation (Fig. 6G) and was accompanied by reduced total IgE serum levels (Fig. 6H), both hallmarks of asthmatic airway inflammation in mice and humans.

To test whether our findings would also have implications for human asthma and/or MS patients, we reanalyzed published data from genome-wide association studies (GWAS). Of note, at least two studies performing GWAS found single-nucleotide polymorphisms (SNPs) in the *MYC* locus to be associated with the risk to develop allergic asthma and MS^36,37^ (https://doi.org/10.1101/195933) (Supplemental Table S1). One study specifically found a SNP within the human *MYC* locus to be associated with a susceptibility to develop allergic asthma already in childhood^37^. Thus, high doses zinc aspartate control MYC-regulated metabolic gene expression and fitness of CD4^+^ T cells and prevent from Th1 central autoimmune inflammation and Th2 asthmatic airway inflammation. In addition, MYC might proof useful as a genetic risk marker for MS and/or asthma.

## Discussion

Autoimmune and allergic diseases including asthma show an increasing incidence^2–4^. The treatment of autoimmune and allergic diseases necessitates a fine balance in inhibiting detrimental immune responses without compromising immunity to pathogens. Despite the development and approval of novel pharmaceuticals including monoclonal antibodies that block signature cytokines of both disease entities, there is still a demand for the identification of safe concomitant treatment regimens.

Here, we set out to systematically investigate the influence of zinc aspartate (UNIZINK), a registered pharmaceutical infusion solution with excellent bioavailability, on murine CD4^+^ T cell gene expression and metabolism. In the studies conducted so far, zinc showed a good safety profile when applied to patients^10–14^.

We found that zinc aspartate dose-dependently (200 > 150 > 100 μM) inhibits the activation, proliferation and Th1 and Th2 differentiation of murine CD4^+^ T cells. Our results and especially the concentrations at which zinc altered CD4^+^ T cell function are in line with previously published reports^9,15,16,20,25^. However, for the first time, we here extend on these studies as we systematically study how higher zinc concentrations affect gene expression and the metabolism of CD3/CD28-activated CD4^+^ T cells. In the presence of zinc several genes important for cell cycle entry and/or progression appeared to be significantly downregulated in CD4^+^ T cells. As a possible reason for this cell cycle arrest, we found genes that encode for glycolytic and TCA cycle enzymes to be significantly downregulated in the presence of zinc. Functionally, this cumulated in an impaired ability to perform glycolysis and OXPHOS. In line with this, higher zinc concentrations impaired glucose uptake and increased mitochondrial ROS production. In this context, several studies have reported that high zinc concentrations induce apoptosis in human and murine T cells^15,25^. We here confirm that higher zinc concentrations reduce the viability of CD4^+^ T cells especially at later time points. Based on our data, we, however, postulate that zinc at first inhibits gene expression of important metabolic enzymes/regulators that are crucial to generate sufficient building blocks for proliferation, a process known as metabolic reprogramming^23,30^, and that cell cycle arrest and apoptosis of CD4^+^ T cells is a secondarily consequence of impaired metabolic reprogramming. Of note, this scenario is supported by data that T cells in the presence of zinc showed reduced gene expression but not increased apoptosis in the first hours following stimulation^25^. On the other hand, we cannot fully rule out that beginning apoptosis at early time points contribute to the here detected transcriptional changes and to impaired glycolysis and OXPHOS at higher zinc concentrations.

The detailed molecular mechanism how high zinc concentrations regulate metabolic gene expression are yet unknown. Zinc, in physiological concentrations, is a trace element that affects several biological and cellular processes^6,7,9^. Supraphysiological zinc levels were shown to inhibit cell proliferation, which was demonstrated to correlate with impaired phosphorylation of ERK and ZAP70 and the induction of p38 phosphorylation^38,39^. These defects in signaling events is in line with our finding of reduced CD44 and CD25 expression in CD4^+^ T cells at higher zinc concentrations.

Recently, the transcription factor FOXO1 was reported to be a negative regulator of T cell metabolism, proliferation and effector function^32,33^, while MYC is a well-known transcriptional regulator of glycolytic genes in T cells, which is in part controlled by FOXO1^34,35^. In our upstream regulator analysis of the DEG controlled by zinc, we detected FOXO1 to be among the most significant upregulated and, inversely, MYC as most significant downregulated transcriptional regulator. Furthermore, upon high zinc concentrations we found significantly reduced nuclear MYC protein expression in T cells. Our data could thus indicate that high zinc concentrations stabilize FOXO1 expression within the nucleus, which secondarily impairs MYC expression and metabolic gene transcription. A possible mechanism for this scenario was recently reported in that the nuclear zinc finger protein ZFAT was demonstrated to maintain FOXO1 protein levels in peripheral T cells^40^.

In our current study, we demonstrate that zinc aspartate prevents Th1 EAE and Th2 asthmatic airway inflammation. It needs to be mentioned that in our in vivo experiments we first activated T cells by a specific antigen (MOG peptide or HDM extract) before zinc was administered to mice during the development of EAE or the rechallenge phase within the HDM asthma model. We decided for this experimental approach as this reflects the treatment situation in the clinical course of MS and asthma patients better as those patients already have developed signs of autoimmunity and asthma symptoms when treatment is initiated. Treating CD4^+^ T cells already in the initial priming phase would have likely evoked similar (or even more significant) protection from EAE and/or asthmatic airway inflammation. It should be further mentioned that zinc administration was shown to promote the formation of inducible regulatory T cells^17,41^ and that this can have contributed to the prevention of EAE and asthmatic airway inflammation in our experiments. In the same vein, zinc was also shown to suppress Th17 cell development^42^. As Th17 cells, besides Th1 cells, are involved in the pathogenesis of MS and EAE, it is likely that impaired Th17 cells, which we have not assed in our experiments, can have contributed to protection from EAE in our study. Besides cells of the adaptive immune system, zinc was shown to affect innate immune cells including macrophages^43^, which are also involved in the pathogenesis of MS and asthma. In the here used mouse models of asthmatic airway inflammation and EAE zinc will also have affected innate immune cells, and this can also have contributed to protection from the diseases.

## Material and methods

### Mice

OT-II TCR transgenic (tg) mice (JAX stock No: 004194) and C57BL/6JRj wild-type (WT) were housed under specific pathogen free (SPF) conditions. For all experiments, mice of both sexes at the age of 8–14 weeks were used.

### Reagents

Zinc aspartate (UNIZINK), a registered pharmaceutical infusion solution with good bioavailability, was purchased from Köhler Pharma GmbH (Alsbach-Hähnlein, Germany).

### Murine CD4^+^ T cell isolation and culture

CD4^+^ T cells were isolated from spleen and cervical, submandibular, brachial, axillar, inguinal, and mesenterial lymph nodes of OT2 TCR tg mice by negative enrichment using the MagniSort mouse CD4^+^ T‐cell enrichment kit (Invitrogen, # 8804-6821-74). Cultures plates were coated with rabbit-anti-hamster IgG (MP Biomedicals, # SKU 0855398) for 2 h at 37 °C. CD4^+^ T cells were stimulated cultured in RPMI medium supplemented with 10% FCS, 2 mM GlutaMAX (ThermoScientific, # 35050038), 50 µM β-mercaptoethanol (ThermoScientific, # 31350010), 2% penicillin/streptavidin (ThermoScientific, # 15070063) and stimulated with 1 μg/ml anti-CD3ε and 1 μg/ml and anti‐CD28 antibodies (BioLegend, # 100340 and 102116).

### Measuring CD4^+^ T cell activation and apoptosis

CD4^+^ T cells were stimulated with 1 μg/ml anti-CD3ε and 1 μg/ml and anti‐CD28 antibodies (BioLegend, # 100340 and 102116) in the absence or presence of 100 μM, 150 μM, 200 μM zinc aspartate for 48 h. To evaluate the activation T cells thereafter were stained for CD4, CD25, CD44 and CD62L and analyzed using an LSR Fortessa flow cytometer (BD Biosciences) and FlowJo v10.7.1 software (BD Biosciences). Early apoptosis was measured after 6 h stimulation in the absence or presence of 50, 100, 150 and 200 μM zinc using AnnexinV staining and flow cytometry. Late apoptosis was determined after 48 h stimulation in the absence or presence of 100, 150 and 200 μM zinc using the APC Annexin V Apoptosis Detection Kit with 7-AAD (BioLegend, # 640930).

### Measuring CD4^+^ T cell proliferation

CD4^+^ T cells were incubated with 5 µM CFSE (Invitrogen, # C34554) for 5 min at room temperature in the dark followed by two washing steps. Thereafter, CD4^+^ T cells were stimulated with 1 μg/ml anti-CD3ε and 1 μg/ml and anti‐CD28 antibodies (BioLegend, # 100340 and 102116) in the absence or presence of 100 µM, 150 µM, 200 µM zinc aspartate. At 48 h and 72 h, CD4^+^ T cells were analyzed for their CFSE dilution by FACS.

### In vitro Th1 and Th2 cell differentiation

For Th1 and Th2 differentiation OT-II TCR tg CD4^+^ T cells were cultured in IgG-coated 12 well plates for 3 days under Th1 or Th2 conditions. For Th1 skewing, cells were cultured in the presence of anti-CD3 (1 µg/ml), anti-CD28 (1 µg/ml), hIL-2 (30 units/ml), anti-IL-4 (2 µg/ml) and IL-12 (10 ng/ml). For Th2 differentiation, cells were cultured in the presence of anti-CD3 (1 µg/ml), anti-CD28 (1 µg/ml), hIL-2 (30 units/ml), anti-IFN-γ (10 µg/ml), anti-Il-12 (10 µg/ml), IL-4 (100 ng/ml). After 3 days, cells were stained for differentiation markers using anti-GATA3-APC, anti-Tbet-PE, anti-IL-4-PE, anti-IFN-γ-APC, anti-TNF-α-APC, anti-IL-2-APC, anti-IL-13-Alexa Flour (488) and either anti-CD4-FITC or anti-CD4-APC antibodies (pl. see Supplemental Table S2). For intracellular staining of cytokines cells were fixed and permeabilized using the eBioscience Intracellular Fixation & Permeabilization Buffer Set (Thermo Scientific #88-8824-00). Transcription factors (TBET, GATA3, MYC) were stained using the eBioscience Foxp3/Transcription Factor Staining Buffer Set (Miltenyi, #130-093-142). Cells were fixed for 45 min at room temperature, centrifuged and resuspended in permeabilization buffer supplemented with the corresponding staining antibodies.

### Seahorse assays

For metabolic seahorse assays, OT-II TCR tg CD4^+^ T cells were stimulated with 1 μg/ml anti-CD3ε and 1 μg/ml and anti‐CD28 antibodies (BioLegend, # 100340 and 102116) in the absence or presence of 100 and 150 µM zinc aspartate for 24 h. CD4^+^ T cell metabolism was analyzed using an XFe96 Extracellular Flux Analyzer (Seahorse Bioscience). 24 h after activation, 2 × 10^5^ CD4^+^ T cells/well were plated in XFe96 cell culture plates coated with Cell-Tak (Fisher Scientific, # 10317081). Seahorse XF DMEM medium was supplemented with 10 mM glucose, 2 mM glutamine and 1 mM pyruvate (MitoStress assay medium, pH adjusted to 7.4) or 2 mM glutamine and 1 mM pyruvate (GlycoStress assay medium, pH adjusted to 7.4). According to the manufacturer’s instruction, OCR was measured in response to 2.5 µM oligomycin, 1.5 µM fluorocarbonyl cyanide phenylhydrazone (FCCP) and 0.5 μM rotenone plus antimycin A (MitoStress Test Kit, Agilent, # 103015-100) and ECAR in response to 10 mM glucose, 2.5 µM oligomycin and 50 mM 2-deoxy-glucose (2-DG) (GlycoStress Test Kit, Agilent, # 103020-100). Each sample was analyzed in technical triplicates and later averaged for independent experiments. Non-mitochondrial oxygen consumption, basal respiration, maximal respiration, proton leak and ATP were determined by monitoring OCR. From ECAR measurements, non-glycolytic acidification, glycolysis, glycolytic capacity, and glycolytic reserve were calculated.

### Glucose uptake and measurement of mitochondrial ROS production

Glucose uptake was measured using 2-NBDG (ThermoFisher Scientific, # N13195). Unstimulated and for 18 h with 1 μg/ml anti-CD3ε and 1 μg/ml and anti‐CD28 antibodies stimulated OT-II TCR tg CD4^+^ T cells were incubated in glucose-free RPMI medium containing 2-NBDG (100 μM) for 90 min in the dark. The amount of 2-NBDG utilized by the cells was determined by FACS. For the measurement of mitochondrial ROS production by FACS, OT-II TCR tg CD4^+^ T cells were incubated with 5 μM MitoSOX™ Red Mitochondrial Superoxide Indicator (ThermoFisher Scientific, # M36008) for 10 min in the dark.

### RNA sequencing and qRT-PCR of CD4^+^ T cells

For bulk RNA sequencing, OT-II TCR tg CD4^+^ T cells were stimulated with 1 μg/ml anti-CD3ε and 1 μg/ml and anti‐CD28 antibodies (BioLegend, #100340 and #102116) in the absence or presence of 100 and 150 µM zinc aspartate for 24 h. RNA was isolated using the Rneasy Plus Mini Kit (QIAGEN, #74136).

Quality and integrity of total RNA was controlled on Agilent Technologies 2100 Bioanalyzer (Agilent Technologies; Waldbronn, Germany). PolyA + RNA were purified from 1 µg total RNA using Dynabeads mRNA DIRECT Micro Kit (ThermoFisher). The RNA Sequencing library was prepared with NEBNext Ultra™ II Directional RNA Library Prep Kit for ILLUMINA (New England Biolabs). The libraries were sequenced on ILLUMINA NovaSeq 6000 using NovaSeq 6000 S1 Reagent Kit (100 cycles, paired end run 2 × 50 bp) with an average of 5 × 107 reads per RNA sample. Each FASTQ file got a quality report generated by FASTQC tool. Before alignment to the reference genome each sequence in the raw FASTQ files was trimmed on base call quality and sequencing adapter contamination using Trim Galore! wrapper tool. Reads shorter than 20 bp were removed from FASTQ file. Trimmed reads were aligned to the reference genome using open source short read aligner STAR (https://code.google.com/p/rna-star/) with settings according to log file. Feature counts were determined using R package “Rsubread”. Only genes showing counts greater 5 at least two times across all samples were considered for further analysis (data cleansing). Gene annotation was done by R package “bioMaRt. Before starting the statistical analysis steps, expression data were log2 transformed and normalized according to 50th percentile (quartile normalization using edgeR). Differential gene expression was calculated by R package “edgeR”. Functional analysis was performed by R package “clusterProfiler” and by using Ingenuity Pathway Analysis (IPA) software (http://www.ingenuity.com/). The lists of DEG containing ENSEMBLE ID and corresponding fold-change values and p/FDR (False Discovery Rate) values, were used as an input for IPA analysis. The curated canonical pathways from Ingenuity Knowledge base were enriched in the DEG dataset. The enrichment p-value with a threshold 0.05 was calculated based on the probability of a pathway being randomly selected from all of the curated pathways. The IPA ‘Diseases and Functions Analysis’ was performed to predict effected biology (cellular process, biological functions) and directional change on that effect. The upstream regulators were predicted based on the expected causal effects between upstream regulators and DEG targets by IPA analysis. Based on the calculation of an overlap p-value and an activation z-score, the analysis gives a prediction of the directional state of the upstream regulator. Lisa analysis (http://lisa.cistrome.org/) was performed to increase confidence in the prediction of the transcriptional regulators of the DEG dataset. For the Heatmap in Fig. 1B, two comparisons were analyzed (100 μM versus 0 μM zinc, 150 μM versus 0 μM zinc) and p-values and false discovery rate (FDR) calculated. Thereafter, genes with the 500 best FDR values were selected and illustrated. The 100 μM condition is not show in the heatmap. KEGG network was created based on the comparison 150 μM versus 0 μM zinc and the resulting gene list when applying a log2FC > 1 or < − 1 with FDR < 0.05.

For qRT-PCR to determine *Myc* gene expression (Fwd: AGTGCTGCATGAGGAGACAC; Rev: GGTTTGCCTCTTCTCCACAG), RNA was isolated using the Rneasy Plus Mini Kit (QIAGEN, #74136) and normalizing to the house keeping gene *Gapdh* (Fwd: TTGATGGCAACAATCTCCAC; Rev: CGTCCCGTAGACAAAATGGT).

### Experimental autoimmune encephalitis

Animals were bred under specific pathogen-free conditions in the central animal facility of the Medical faculty of the Otto-von-Guericke University Magdeburg. All procedures were approved by local government agencies (Landesverwaltungsamt Sachsen-Anhalt, 42502-2-1273 Uni MD, 17.11.2014). Passive induction of EAE by adoptive transfer of polarized MOG-specific T cells (TCR Vα3.2 Vβ11) was performed as previously described (Engelmann et al., 2013^44^). Briefly, splenocytes from 2D2 mice were cultured and stimulated with MOG_35-55_ (20 ng/ml) in the presence of 10 ng/ml IL 2 and IL-7 (Miltenyi Biotech, 130-120-662; 130-094-066) for 2 days. At the end of this incubation period, cells were expanded with IL-2 and IL-7 for another 4 days. Subsequently, cells were reactivated for 24 h with plate-bound anti-CD3 and anti-CD28 antibodies (1 µg/mL) in the presence of 20 ng/ml IL-12 and IL-18 (Bio-techne Ltd., 419-ML-010; 9139-IL-010). Activated T cells were collected, washed and 4–5 × 10^6^ cells in PBS were i.p. transferred into recipient mice. Successful activation and polarization was monitored via flow cytometric analysis of CD62L shedding and CD69 upregulation on TCR Vα3.2^+^ Vβ11^+^ CD4^+^ T cells. Zinc aspartate was administered daily i.p. (15 µg/animal) starting at day 8 after the appearance of first clinical signs. Zinc treatment was continued for 7 days. The control group received PBS. Mice were examined daily for signs of disease and graded on a scale of increasing severity from 0 to 5 as follows: 0, no signs; 0.5, partial tail weakness; 1, limp tail or slight slowing of righting from supine position; 1.5, limp tail and slight slowing of righting; 2, partial hind limb weakness or marked slowing of righting; 2.5, dragging of hind limb(s) without complete paralysis; 3, complete paralysis of at least one hind limb; 3.5, hind limb paralysis and slight weakness of forelimbs; 4, severe forelimb weakness; 5, moribund or dead^45^. For reasons of animal welfare, mice were killed when reaching a score of 3 or above. Mean clinical scores were calculated by adding disease scores of individual mice divided by the number of mice in each group.

### House dust mite induced allergic airway inflammation

C57BL/6JRj wild-type (WT) mice were anesthetized with isoflurane and intranasally (i.n.) sensitized with 10 µg house dust mite extract (HDM) (Citeq Biologics, 02.01.85, Batch 18G08) for three consecutive days (days 0, 1, and 2). Rechallenges with 10 µg HDM extract were performed for four days (days 10, 11, 12, and 13). On days 10, 11, 12 and 13, part of the mice received intraperitoneally (i.p.) 30 µg UNIZINK (Köhler Pharma GmbH), while control mice received PBS. On day 14, mice of both groups were euthanized and blood was collected, which was later analyzed for total IgE levels using an IgE ELISA kit (Thermo Scientific, # 88-50460-86). In addition, parts of the lungs were fixed with 4% formaldehyde and later analyzed for peribronchial airway inflammation by HE staining. Remaining lung tissue was weighted and thereafter enzymatically digested with tissue Liberase TL (Roche, # 05 401 020 001) in RPMI 10% FCS, 2 mM GlutaMAX, 50 µM β-mercaptoethanol, 2% penicillin/streptomycin at 37 °C for 45 min. Afterwards single cell suspensions from lungs and mediastinal lymph nodes were filtered using a 70 μm cell strainer. Part of the cells of lungs and mediastinal lymph nodes were stained for CD45 and CD4 and then permeabilized using the Foxp3 / Transcription factor staining buffer set (Miltenyi, #130-093-142) followed by intracellular GATA-3 staining and FACS analysis. Another part of the single cell suspensions of the lungs and mediastinal lymph nodes were restimulated with 1 µM ionomycin (ThermoFisher Scientific, # A1049201) and 20 nM PMA (Sigma-Aldrich, #P1585-1MG) in the presence of 5 µM Brefeldin A (Biolegend, # 420601) in RPMI1640 supplemented with 10% FCS, 2 mM GlutaMAX, 50 µM β-mercaptoethanol, 2% penicillin/streptomycin for 6 h at 37 °C. Restimulated cell suspensions were stained for CD45 and CD4, fixed and permeabilized using the eBioscience™ Intracellular Fixation & Permeabilization Buffer Set (ThermoScientific, # 88-8823-88) and intracellularly stained for IL-4, IL-13 and TNFα before FACS analysis. To account for reported sex differences in the murine HDM asthmatic airway inflammation model female mice were used in both groups^46,47^. All procedures were approved by local government agencies (Landesverwaltungsamt Sachsen-Anhalt, 42502-2-1633 UniMD).

### Scoring of the HE stained lungs and IgE ELISA

Lung tissues were fixed in 4% buffered formaldehyde for at least 24 h. Paraffin-embedded lung sections of 2 µm were stained with haematoxylin and eosin (HE). Representative sections (2 per animal) were chosen and the amount of bronchiole-surrounding, inflammatory cells was analyzed. The peribronchial cellular infiltration was finally calculated as semi-quantitative score (0—0% surrounding cells, 1—< 10% surrounding cells, 2—10–25% surrounding cells, 3—25–50% surrounding cells, 4—> 50% surrounding cells)^48,49^. Serum was collected from whole blood samples after incubation for 30 min at room temperature (RT) and subsequent centrifugation at 300×*g* for 10 min. Serum total IgE levels were analyzed with IgE Mouse Uncoated ELISA Kit (ThermoFisher Scientific, # 88-50460). Serum samples were pre-diluted 25-fold in Assay Buffer A and processed according to manufacturer’s protocol.

### Ethics declaration

All procedures were approved by local government agencies to S.K. for the HDM model (Landesverwaltungsamt Sachsen-Anhalt, 42502-2-1633 UniMD) and to A.R. for the EAE model (Landesverwaltungsamt Sachsen-Anhalt, 42502-2-1273 Uni MD, 17.11.2014), respectively. All methods were carried out in accordance with relevant guidelines and regulations and are reported in accordance with ARRIVE guidelines (www.arriveguidlines.org).

### Statistical analysis

All data were analyzed using GraphPad Prism 8.0 (GraphPad software, Inc.) and are presented as mean ± SEM, if not indicated otherwise. Comparisons across multiple groups were performed by ordinary one-way ANOVA. Experiments with two groups were analyzed with Student’s t-test. Statistical comparison of the mean EAE score between two different groups of animals was accomplished by performing non-parametric Wilcoxon matched pairs test using GraphPad Prism software (GraphPad software, Inc.). P values < 0.05 were considered as statistically significant; different levels of significance were indicated as follows: *P < 0.05; **P < 0.01; ***P < 0.001.

## Supplementary Information

Supplementary Information.
