# Supplementary material for: Ionic mitigation of CD4+ T cell metabolic fitness, Th1 central nervous system autoimmunity and Th2 asthmatic airway inflammation by therapeutic zinc
Source: Sci Rep. 2022 Feb 4;12:1943. doi: 10.1038/s41598-022-04827-6 (PMC8816938; doi:10.1038/s41598-022-04827-6)
Supplement: Supplementary file 1 — Supplementary Information. [file 41598_2022_4827_MOESM1_ESM.pdf]

**Supplementary information**

**Supplemental Table 1: GWAS identify MYC as potential risk gene for the development of allergic asthma and multiple sclerosis.**

Allergic asthma

| Gene | SNP | OR | p-value | Publication |
|---|---|---|---|---|
| MYC | rs13277355 | 1.11 | 1.65x10-10 | Pividori, M. et al., 2019 |

Multiple sclerosis

| Gene | SNP | OR | p-value | Publication |
|---|---|---|---|---|
| MYC | rs4410871 | 1.09 | 7.70 x10-09 | Gourraud et al., 2012 |

**Supplemental Table 2: Antibodies used for FACS analyses**

| Antigen | Clone | Company | Fluorochrom |
|---|---|---|---|
| CD62L | MEL-14 | BioLegend | AlexaFluor488 |
| CD44 | IM7 | BioLegend | PE |
| CD25 | 3C7 | BioLegend | FITC |
| CD44 | IM7 | BioLegend | FITC |
| CD4 | RM4-5 | BioLegend | APC |
| CD45 | 30-F11 | BioLegend | BV570 |
| CD4 | RM4-5 | BioLegend | PacificBlue |
| PD-1 | 29F.1A12 | BioLegend | PE |
| CXCR5 | L138D7 | BioLegend | PE/Cy7 |
| B220 | RA3-6B2 | BioLegend | FITC |
| GATA3 | 16E10A23 | BioLegend | APC |
| IL-4 | 11B11 | BioLegend | PE-Cy7 |
| IL-4 | 11B11 | BioLegend | PE |
| IL-13 | eBio13A | ThermoScientific | AF488 |
| TNFα | MP6-XT22 | BioLegend | APC |
| MYC | D84C12 | Cell Signaling | N/A |

# Suppl. Figure 1

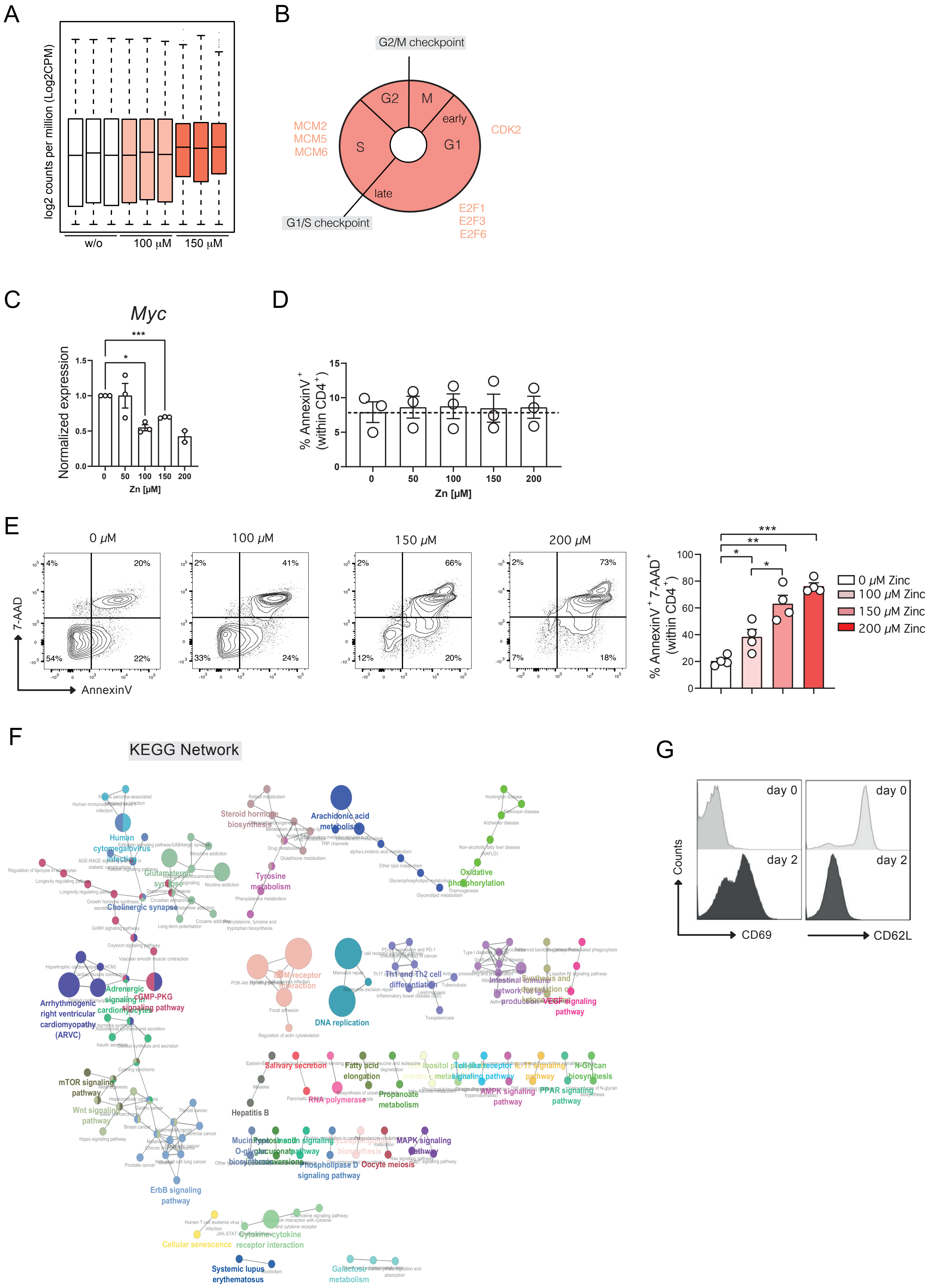

**Suppl. Figure 1: High dose zinc aspartate controls gene expression and metabolic fitness of $CD4^+$ T cells.**

A, Boxplot showing log2 counts per million of the individual biological samples for RNA seq.

B, Cell cycle phases with the indicated genes out of Figure1E.

C, OT-II TCR tg $CD4^+$ T cells were activated for 6 hrs in the absence and presence of the indicated zinc concentrations. Graphs show normalized gene expression of *Myc* determined by qRT-PCR and using the housekeeping genes *Gapdh*. Data are from 3 mice (except for 200 μM, here 2 mice were analyzed).

D, OT-II TCR tg $CD4^+$ T cells were activated for 6 hrs in the absence and presence of the indicated zinc concentrations and analyzed for early apoptosis using AnnexinV staining and flow cytometry. Data are from 3 mice.

E, OT-II TCR tg $CD4^+$ T cells were activated for 48 hrs in the absence and presence of the indicated zinc concentrations and analyzed for cell death using AnnexinV and 7-AAD staining and flow cytometry. Data are from 4 mice.

F, KEGG network analysis of bulk RNA sequencing data as shown in Figure 1.

G, Representative stainings for CD69 and CD62L of 2D2 TCR tg T cells before and after activation as described in Figure 5E.

RNA sequencing data in A was generated in biological triplicates from 3 mice per group. Data in C and D are from 3 mice. Data in E are from 4 mice. Every dot represents one mouse. Statistical analysis in C-E by one-way ANOVA followed by Tukey's honestly significant difference (HSD) post hoc test for multiple comparisons. p*<0.05, **p<0.01, ***p<0.001.
